# Prevalence of vitamin D deficiency in exclusively breastfed infants at a tertiary healthcare facility in Nairobi, Kenya

**DOI:** 10.20945/2359-3997000000281

**Published:** 2020-08-24

**Authors:** Nusrat Abubakar Said, Rose Wanjiru Kamenwa, Mary Slessor Limbe, Mitchel Otieno Okumu, William Maina Macharia

**Affiliations:** 1 The Aga Khan University Hospital Department of Pediatrics and Child Health Nairobi Kenya Department of Pediatrics and Child Health, The Aga Khan University Hospital, Nairobi, Kenya; 2 Avenue Hospital Kisumu Department of Pediatrics Kisumu Kenya Department of Pediatrics, Avenue Hospital Kisumu, Kisumu, Kenya; 3 Referral Hospital Jaramogi Oginga Odinga Teaching Department of Pharmacy Kisumu Kenya Department of Pharmacy, Jaramogi Oginga Odinga Teaching, and Referral Hospital, Kisumu, Kenya; 4 University of Nairobi Department of Public Health, Pharmacology, and Toxicology Nairobi Kenya Department of Public Health, Pharmacology, and Toxicology, University of Nairobi, Nairobi, Kenya

**Keywords:** 25OHD, vitamin D, vitamin D deficiency, parathyroid hormone, vitamin D insufficiency, exclusively breastfed infants

## Abstract

**Objective:**

To determine the prevalence of vitamin D deficiency (VDD) in exclusively breastfed infants at the Aga Khan University Hospital Nairobi, Kenya (AKUHN). The relationships between 25-hydroxyvitamin D; 25OHD, parathyroid hormone (PTH), maternal vitamin D supplementation, and sunlight exposure were also determined.

**Subjects and methods:**

Blood from 98 infants was assayed for 25OHD, calcium, phosphate, and PTH. Socio-demographic and clinical characteristics were analyzed using descriptive statistics and inferential analysis ( *p* < 0.05).

**Results:**

The prevalence of VDD (25OHD <12 ng/mL), vitamin D insufficiency (VDI, 25OHD 12-20 ng/mL) and vitamin D sufficiency (VDS, 25OHD >20 ng/mL) was 11.2% (95% CI 8.0%-14.4%), 12.2% (95% CI 8.9%-15.5%), and 76.5% (95% CI 72.3%-80.8%) respectively. There was no difference in the mean age, head circumference, length, or weight of infants in VDD, VDI, and VDS groups. PTH was elevated when 25OHD was <12 ng/mL and normal when 25OHD was between 12-20 ng/mL. 25OHD and PTH were normal in infants whose mothers received vitamin D supplements. Infants who received <30 minutes/day of exposure to sunlight were 5 times more likely to have VDI than infants who received ≥30 minutes/day ( *p* = 0.042).

**Conclusions:**

The prevalence of VDD in exclusively breastfed infants at AKUHN is low. The current national policy that recommends exclusive breastfeeding of infants in the first 6 months of life appears to be effective in staving off vitamin D deficiency but those infants with < 30 minutes sunlight exposure may benefit from low dose supplemental vitamin D during times of low sunlight exposure.

## INTRODUCTION

Vitamin D is a fat-soluble steroid hormone that is produced when the skin is exposed to sunlight ( 1 ). It is involved in the reduction of inflammation, modulation of cell growth, normal functioning of the nervous and immune systems, and bone metabolism ( 2 ). The human body obtains >80% of vitamin D when ultraviolet-B radiation (UV-B) converts 7-dehydrocholesterol in epidermal and dermal cells to 25-hydroxyvitamin D; 25OHD ( 3 , 4 ). This metabolite is subsequently hydroxylated in the liver and kidney to form 1,25-dihydroxyvitamin D; 1,25(OH)_2_D ( 3 , 4 ). Vitamin D (∼20%) may also be supplied via the diet ( 3 , 4 ).

25OHD has a long half-life in the circulation and its concentration is not under tight homeostatic regulation ( 5 , 6 ). It is therefore useful for measuring the levels of vitamin D in the human body ( 5 , 6 ). It may be assayed by chromatographic, spectroscopic, or immunosorbent techniques including high-pressure liquid chromatography, liquid chromatography-tandem mass spectroscopy, radioimmunoassays, and competitive protein binding assays ( 7 ). The analysis and interpretation of 25OHD is made by using thresholds of <12 ng/mL, 12-20 ng/mL, and >20 ng/mL to denote vitamin D deficiency, vitamin D insufficiency and vitamin D sufficiency respectively in keeping with the recommendations of several global health authorities including the American Institute Of Medicine (IOM), the UK Scientific Advisory Committee on Nutrition (SACN), the Nordic Nutritional Recommendations (NNR), and the European Food Safety Authority (EFSA) ( 8 – 11 ).

Severe VDD causes osteomalacia in adults and rickets in children ( 12 – 14 ). However, because vitamin D is physiologically involved in other body systems, health impacts such as osteoporosis, breast cancer, and preeclampsia may also be realized ( 12 – 14 ). Estimates suggest that up to a billion people are affected by VDD and VDI worldwide ( 15 ). Exclusively breastfed infants are particularly vulnerable to VDD and maternal VDD during pregnancy has been identified as one of the major contributors of VDD in infants ( 16 ). For example, at AKUHN, eight out of every ten-term newborn infants are born to vitamin-D-deficient mothers ( 16 ).

The National Nutrition Action Plan is a policy in Kenya that recommends the exclusive breastfeeding of infants until they are six months of age ( 17 ). While breastfeeding is the recommended method of infant feeding and provides infants with the necessary nutrients and immune factors, breast milk provides only about 10-80 IU/L of vitamin D which is short of the recommended 400 IU and is likely to result in VDD ( 18 ). By evaluating the prevalence of VDD among exclusively breastfed infants in the region, the scope of VDD as a public health problem can be realized. Presently, this data is not available. This study, therefore, aims to fill this gap by determining the prevalence of VDD among exclusively breastfed infants at AKUHN, a private referral hospital in Nairobi, Kenya. The relationship between 25OHD, PTH, maternal vitamin D supplementation, and sunlight exposure was also determined.

## SUBJECTS AND METHODS

### Study setting

The Aga Khan University Hospital (AKUHN) is a private referral hospital that serves residents of Nairobi and the wider East African region ( 19 ). This institution offers both outpatient and inpatient pediatric services ( 19 ). A well-baby clinic is also available which provides immunization services and growth monitoring for infants and children under the age of five years ( 19 ). On average, 30 infants aged three to six months are seen at the hospital per week ( 19 ).

### Ethical considerations

Ethical approval was sought from the ethics committee at the Aga Khan University Hospital; AKUHN (REF: 2014/REC-23 (v2); Supplementary data; Appendix I). The purpose of the study was carefully explained to all the mothers of participating infants and an opportunity was provided to them to seek clarifications regarding the study. Mothers were requested to sign an informed consent form detailing the terms of participation (Supplementary data; Appendix II). Mothers were free to withdraw their child from the study at any time. In keeping with the guidelines of the Department of Pediatrics at AKUHN, routine blood sampling was usually done for all infants at six months of age to determine their hemoglobin levels. For these infants, sampling for this study was done at the same time to avoid multiple pricks. EMLA^®^ (lidocaine) cream, a topical anesthetic agent was applied at the sampling site to minimize pain. Tests were carried out at no cost to the patients. Confidentiality was maintained by using codes instead of personal identifying information.

### Study design

**Figure 1:**
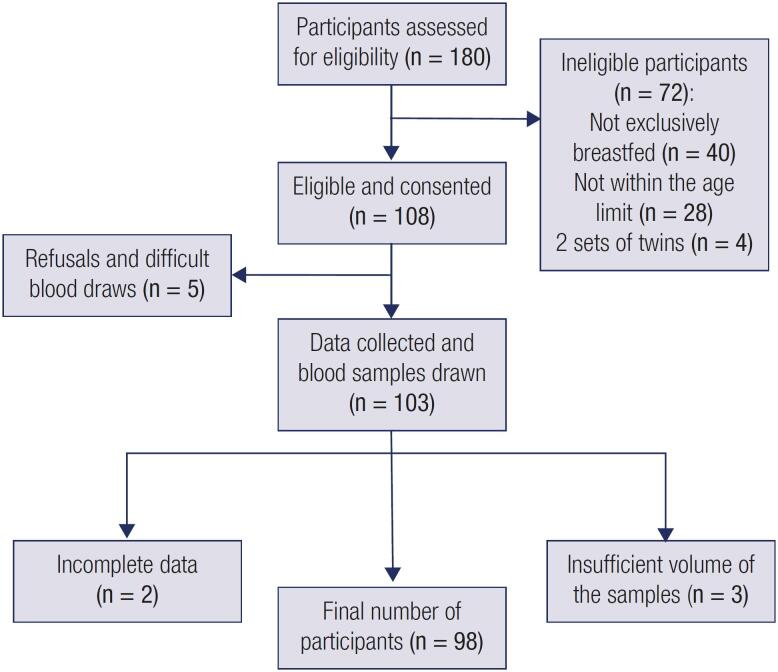
Flow chart showing the criteria of recruiting infants at the Aga Khan University Hospital Nairobi, Kenya.

This was a cross-sectional study to determine the prevalence of vitamin D deficiency in exclusively breastfed infants at the Well-Baby Clinic of the Aga Khan University Hospital, Nairobi (AKUHN) between October 2014 and April 2015. Infants who fit the following characteristics were included in the study: a) term (>37 weeks gestational age at birth) singleton infants aged three to six months old, b) exclusively breastfed infants, and c) informed consent was obtained from the parent to participate in the study. Preterm infants, those receiving Vitamin D supplements, those with kidney/liver, parathyroid or bone diseases or malabsorption syndromes and infants on medication known to alter vitamin D metabolism (anticonvulsants, anti-tubercular drugs, antifungals, and antiretrovirals) were excluded from the study. A flow chart showing the criteria of recruiting infants at the Aga Khan University Hospital Nairobi, Kenya is summarized in Figure 1 .

### Sample size estimation

Fischer's formula was used to calculate the sample size ( 20 ).


$$\text{i}.\text{e}.\text{N}=\frac{{\text{Z}}^{2}\text{ P }(1-\text{P})}{{\text{D}}^{2}}$$

Where;

N = minimum sample size

Z = standard normal deviation corresponding to 95% confidence interval (= 1.96) in a 2-tailed test.

P = the expected proportion (50%)

D = degree of precision (10%)

N = 96

The expected proportion was estimated at 50% to give the largest sample size with a 10% degree of precision. The choice of the degree of precision value of 10% was due to financial and time limitations.

### Sampling technique

All three to six-month-old infants visiting the well-baby clinic from October 2014 were evaluated for eligibility. Recruitment continued until the desired sample size was achieved; April 2015. A focused physical examination was done on each of the recruited infants to assess for skeletal signs of rickets. The outcome of the examination was recorded in a pre-designed form (Supplementary data; Appendix III) that was coded for each participant. This remained in the custody of the principal investigator. The phlebotomy site was identified, swabbed with alcohol, and a sample of 2 mL of blood was drawn from a vein and collected in standard sampling tubes (clear red-topped vacutainers). The specimen bottle for each participant bore the same unique code as the recruitment form with the patient's details. Blood samples were sent to the laboratory and analyzed daily.

### ELECSYS 25OHD total assay

#### Reagents – Working solutions

The reagent rack pack (M, R1, and R2) and the pretreatment reagents (PT1, and PT2) were labeled as VITD-T. PT1 Pretreatment reagent 1 (white cap), 1 bottle, 4 mL: Dithiothreitol 1 g/L, pH 5.5. PT2 Pretreatment reagent 2 (gray cap), 1 bottle, 4 mL: Sodium hydroxide 55 g/L. M Streptavidin-coated microparticles (transparent cap), 1 bottle, 6.5 mL: Streptavidin-coated microparticles 0.72 mg/mL; preservative. R1 Vitamin D binding protein-BPRu (gray cap), 1 bottle, 9 mL: Ruthenium labeled vitamin D binding protein 150 μg/L; bis-tris propane buffer 200 mmol/L; albumin (human) 25 g/L; pH 7.5; preservative. R2 25-hydroxyvitamin D∼biotin (black cap), 1 bottle, 8.5 mL: Biotinylated vitamin D (25-OH) 14 μg/L; bis-tris propane buffer 200 mmol/L; pH 8.6; preservative ( 21 ).

#### Assay

A Cobas analyzer (Roche, Mannheim, Germany) was used for these measurements according to manufacturers’ protocols ( 21 ). The assay involved a 3-step incubation process, over a 27-minute duration ( 21 ). In the first step, the sample was incubated with pretreatment reagent, which released bound 25-OH vitamin D from the vitamin D binding protein (VDBP) ( 21 ). In step 2, the pretreated sample was incubated with ruthenium labeled VDBP creating a complex between the 25-OH vitamin D and the ruthenylated VDBP ( 21 ). The third incubation step involved the addition of streptavidin-coated microparticles and 25-OH vitamin D labeled with biotin ( 21 ). The free sites of the ruthenium labeled VDBP become occupied, forming a complex consisting of the ruthenium labeled vitamin D binding protein and the biotinylated 25-OH vitamin D ( 21 ). The entire complex then becomes bound to the solid phase via interaction of biotin and streptavidin ( 21 ). The reaction mixture was then aspirated into the measuring cell where the microparticles were magnetically captured onto the surface of the electrode ( 21 ). Unbound substances were then removed with ProCell/ProCell M. Application of a voltage to the electrode induced a chemiluminescent emission which was measured by a photomultiplier ( 21 ). Results were determined via a calibration curve which was instrument specifically generated by 2-point calibration and a master curve provided via the reagent barcode ( 21 ). 25OHD was reported in nanograms per milliliter (ng/mL) ( 21 ).

### ELECSYS PTH assay

#### Reagents – Working solutions

The reagent rack pack was labeled as PTH ( 22 ). Streptavidin-coated microparticles (transparent cap), 1 bottle, 6.5 mL: Streptavidin-coated microparticles 0.72 mg/mL; preservative. R1 Anti-PTH Ab∼biotin (gray cap), 1 bottle, 7 mL: Biotinylated monoclonal anti-PTH antibody (mouse) 2.3 mg/L; phosphate buffer 100 mmol/L, pH 7.0; preservative. R2 Anti-PTH-Ab∼Ru(bpy) (black cap), 1 bottle, 7 mL: Monoclonal anti-PTH antibody (mouse) labeled with ruthenium complex 2.0 mg/L; phosphate buffer 100 mmol/L, pH 7.0; preservative ( 22 ).

#### Assay

A Cobas analyzer (Roche, Mannheim, Germany) was used for these measurements according to manufacturers’ instructions ( 22 ). The assay involved a 2-step incubation process, over an 18-minute duration of time ( 22 ). Step 1 involved incubating 50 μL of the sample, a biotinylated monoclonal PTH-specific antibody, and a monoclonal PTH-specific antibody labeled with a ruthenium complex to form a sandwich complex ( 22 ). Step 2 involved the addition of streptavidin-coated microparticles, and the complex became bound to the solid phase via the interaction of biotin and streptavidin ( 22 ). The reaction mixture was then aspirated into the measuring cell where the microparticles were magnetically captured onto the surface of the electrode ( 22 ). Unbound substances were then removed with ProCell/ProCell M. Application of a voltage to the electrode induced chemiluminescent emission which was measured by a photomultiplier ( 22 ). Results were determined via a calibration curve which was instrument generated by 2-point calibration and a master curve provided via the reagent barcode ( 22 ). PTH levels were reported as picograms/milliliter (pg/mL) ( 22 ).

### Calcium and phosphate assay

A Cobas analyzer (Roche, Mannheim, Germany) which uses the electrochemiluminescence (ECLIA) assay principle was used for these measurements ( 23 , 24 ). The precision of this analyzer is summarized in (Appendix VI and VII).

### Data handling and analysis

The collected data were entered into Microsoft Excel^®^. 25OHD, calcium, phosphate, and PTH were analyzed by descriptive statistics. Proportions were used in the analysis of vitamin D prevalence and the mean levels PTH, calcium, and phosphate in the VDD, VDI, and VDS groups were compared by One Way Analysis of Variance (ANOVA) and Bonferroni test as post hoc test. The relationship between PTH and 25OHD was determined by scatter plot analysis and odds ratio was used to determine the relationship between infant serum 25OHD and maternal vitamin D supplementation and sunlight exposure. *p* <0.05 was considered significant in all cases. GenStat 15^th^ edition and SPSS version 20.0 were used.

## RESULTS

### Socio-demographics of the study participants

The mean age of the recruited infants was 4.6 months. 54.1% (53/98) were male and 45.9% (45/98) were female.

### Prevalence of vitamin D deficiency

The prevalence of vitamin D deficiency (25OHD <12 ng/mL) among exclusively breastfed infants at AKUHN was 11.2% (95% CI: 8.0%-14.4%), and 12.2% (95% CI: 8.9%-15.5%) were found to have insufficient levels (12 ng/mL to 20 ng/mL) while 76.5% (95% CI: 72.3%-80.8%) had a sufficient level

### Relationship between 25OHD, phosphate, calcium, and parathyroid hormone (PTH)

**Figure 2:**
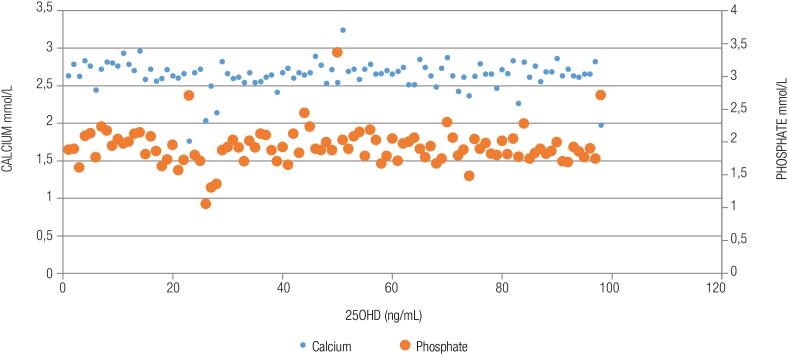
Relationship between 25OHD and serum calcium and phosphate in infants at the Aga Khan University Hospital Nairobi, Kenya.

**Figure 3:**
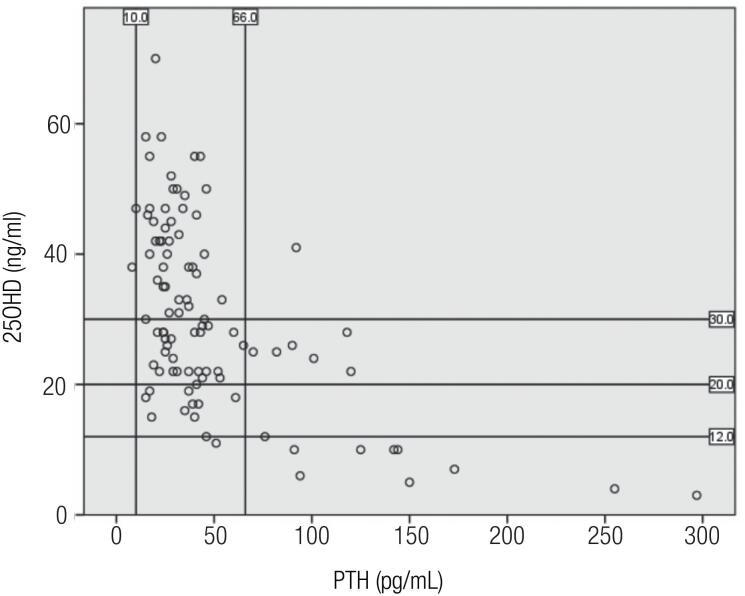
Relationship between 25OHD and PTH in infants at the Aga Khan University Hospital Nairobi, Kenya.

Table 1 is a summary of the mean values of sociodemographic and clinical variables of infants at the Aga Khan University Hospital, Nairobi. There was no significant difference in the mean age, head circumference, length, or weight of infants in the VDD, VDI, and VDS groups ( Table 1 ). The mean phosphate level in the VDD group was significantly ( *p* <0.05) lower than the mean levels in the VDI and VDS groups ( Table 1 ). The mean parathyroid level in the VDD group was significantly ( *p* <0.05) higher than the mean levels in the VDI and VDS groups ( Table 1 ). There was no significant difference in the mean calcium levels of VDD, VDI, and, VDS groups ( Table 1 ). There was a positive and direct relationship between 25OHD and calcium and phosphate ( Figure 2 ). This relationship was more discernible at 25OHD < 30 ng/mL ( Figure 2 ). PTH was mostly elevated when 25OHD was < 12 ng/mL ( Figure 3 ). PTH was normal when 25OHD was between 12 ng/mL to 20 ng/mL ( Figure 3 ). However 6 infants had elevated PTH despite having 25OHD between 20 and 30 ng/mL ( Figure 3 ).

**Table 1:** Summary of the mean values of sociodemographic and clinical variables of infants at the Aga Khan University Hospital

Variable	VDD (n:11)	VDI (n:12)	VDS (n:75)
**Socio demographic variables**			
Age (months)	4.3 (1.2)^a^	4.2 (0.8)^a^	4.8 (1.0)^a^
Head circumference (cm)	43.7 (1.4)^a^	42.5 (0.7)^a^	43.0 (1.8)^a^
Length (cm)	63.0 (2.2)^a^	62.5 (2.0)^a^	63.4 (3.5)^a^
Weight (cm)	7.6 (1.2)^a^	6.8 (0.7)^a^	7.4 (1.2)^a^
**Clinical variables**			
Calcium (mmol/L)	2.5 (0.2)^a^	2.6 (0.2)^a^	2.7 (0.2)^a^
Phosphate (mmol/L)	1.6 (0.2)^a^	2.0 (0.5)^b^	2.0 (0.2)^b^
25OHD (ng/mL)	7.9 (3.1)^a^	16.8 (2.3)^a^	35.8 (11.3)^b^
PTH (pg/mL)	145.3 (74.6)^b^	35.5 (13.9)^a^	37.4 (22.8)^a^

Values expressed as means and standard deviation. One-Way ANOVA and Bonferroni post hoc test was used. Means with different superscripts across the rows are significantly different at *p* <0.05. VDD: vitamin D deficiency; VDI: vitamin D insufficiency; VDS: vitamin D sufficiency.

### Maternal vitamin D supplementation and rachitic manifestations in infants

**Figure 4:**
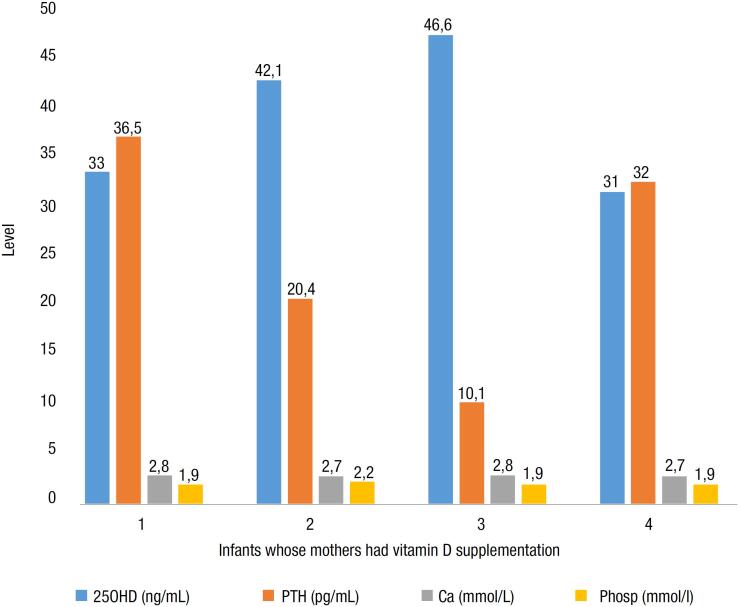
25OHD, PTH, calcium, and phosphate in infants at the Aga Khan University Hospital, Nairobi, Kenya whose mothers had vitamin D supplementation.

**Figure 5:**
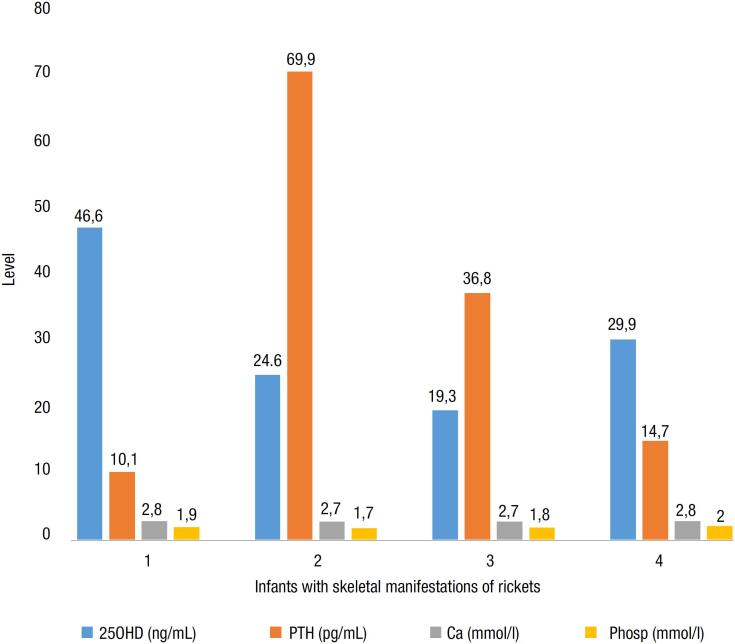
Metabolic features of infants with skeletal manifestations of rickets at the Aga Khan University Hospital Nairobi, Kenya

There were four infants whose mothers were on vitamin D supplementation ( Figure 4 ). All of these infants had sufficient 25OHD and none had elevated PTH. About 4/98 (4.1%) of the infants manifested skeletal signs of rickets. Figure 5 is a summary of the metabolic features of infants with skeletal manifestations of rickets.

### Relationship between 25OHD and sunlight exposure in infants and mothers

Infants with <30 minutes of exposure to sunlight were 5 times more likely to have vitamin D insufficiency than infants who received ≥30 minutes of exposure to sunlight ( *p=* 0.042) – Table 2 .

**Table 2:** Relationship between exposure to sunlight in infants and mothers and 25OHD in infants at the Aga Khan University Hospital Nairobi, Kenya

Optimal exposure to sunlight						
	Infants			Mothers		
Status	Odds ratio	Confidence intervals	*p* value	Odds ratio	Confidence intervals	*p* value
VDS	1.0	−	−	1.0	−	−
VDI	4.98	1.06-23.36	0.04 *	0.63	0.15-2.61	0.53
VDD	1.35	0.31-5.73	0.53	0.66	0.15-2.82	0.58

VDS: vitamin D sufficient; VDI: vitamin D insufficient; VDD: vitamin D deficient.

* Significant at *p* <0.05.

## DISCUSSION

To the best of our knowledge, this is the first study to determine the prevalence of VDD in exclusively breastfed infants in the study area. Considering that the region receives adequate UV radiation throughout the year ( 25 ) this prevalence is somewhat unexpected. It is plausible that this finding may have something to do with the fact that some of the participating infants had been born during or before the 3 coldest months of the year i.e. July, August, and September when temperatures were as low as 9 °C ( 26 ). There is a tendency for mothers to wrap their babies in multiple layers of clothing during harsh (cold) weather conditions to keep them warm. This is particularly true when the infants are outdoors. Therefore, not only is infant sun exposure occluded but also the cutaneous synthesis of vitamin D is impaired.

The prevalence rate we have reported was lower than what was reported in a similar study at the Rubaga hospital in Kampala, Uganda ( 27 ). This observation may be the result of differences in policy between the two hospitals. Sun exposure is impressed upon mothers at discharge from the postnatal unit of AKUHN and every well-baby clinic visit thereafter. This is in light of findings by Dodia and cols. who observed that there was a high prevalence of VDD in newly delivered mothers at AKUHN ( 16 ). Through this study, the cut-off value for VDD in the study area has been determined for the first time. The value is within the clinical threshold set by various global health authorities including the American Institute of Medicine (IOM), the UK Scientific Advisory Committee on Nutrition, the Nordic Nutritional Recommendations, and the European Food Safety Authority ( 8 – 11 ).

We observed that infants whose mothers had received vitamin D supplementation had normal levels of calcium, phosphate, and 25OHD ≥30 ng/mL. This suggests that maternal vitamin D supplementation during pregnancy and breastfeeding may be beneficial in staving off vitamin D deficiency in exclusively breastfed infants.

The finding that even the severely vitamin D-deficient infants had serum calcium and phosphate levels that were within normal reference limits supports the specification of vitamin D deficiency being at 25OHD <12 ng/mL. The reference values for parathyroid hormone (PTH) in infants is between 9.4 and 66 ng/mL ( 28 ). On visual inspection parathyroid hormone (PTH) was elevated when 25OHD was below 12 ng/mL, and PTH were all normal when 25OHD was between 12 ng/mL to 20 ng/mL. Active vitamin D (calcitriol) facilitates the absorption of calcium and phosphorous from the gut and its deficiency reduces calcium absorption and serum calcium levels, which triggers greater PTH synthesis ( 28 ). PTH elevation was observed in 6 infants who had 25OHD between 20 and 30 ng/mL and normal calcium and phosphate. Possible explanations may be that there may have been low maternal calcium intake or that the infants may have had some renal impairment/malabsorption syndromes. However, we believe that the reduced maternal calcium may be a more likely explanation for the PTH elevation. This is because not only was there no stunted development of observed in infants during physical examination but also part of our infant recruitment strategy involved excluding infants with kidney/renal impairment, malabsorption syndromes, and those on medication known to alter vitamin D metabolism i.e. anticonvulsants, anti-tubercular drugs, antifungals, and antiretrovirals).

Less than 5% of study participants had signs of rickets (wrist prominence, Harrison's sulcus, and a rachitic rosary). This observation, when taken in the context of the 11.2% of infants who had VDD and the 12.2% of infants who had VDI suggests that skeletal features may be late manifestations of vitamin D deficiency and therefore there may be a need to review the use of these features in informing clinical decisions relevant to vitamin D deficiency.

In conclusions, our findings suggest that the prevalence of VDD among exclusively breastfed infants in the study area is low. The current policy that recommends the exclusive breastfeeding of infants in Kenya until they are six months of age appears to be effective in staving of vitamin D deficiency and should be promoted in hospitals with similar infrastructure as Aga Khan University Hospital, Nairobi. Because of the higher risk of vitamin insufficiency in infants with <30 minutes of sunlight exposure, low dose supplemental vitamin D during times of low sunlight exposure may be recommended.
